# Analogues of 2′-hydroxychalcone with modified C4-substituents as the inhibitors against human acetylcholinesterase

**DOI:** 10.1080/14756366.2020.1847100

**Published:** 2020-11-26

**Authors:** Sri Devi Sukumaran, Shah Bakhtiar Nasir, Jia Ti Tee, Michael J. C. Buckle, Rozana Othman, Noorsaadah Abd. Rahman, Vannajan Sanghiran Lee, Syed Nasir Abbas Bukhari, Chin Fei Chee

**Affiliations:** a Faculty of Medicine, Department of Pharmacy, University of Malaya, Kuala Lumpur, Malaysia; b Faculty of Science, Department of Chemistry, University of Malaya, Kuala Lumpur, Malaysia; c College of Pharmacy, Jouf University, Al-Jouf, Kingdom of Saudi Arabia; d Nanotechnology and Catalysis Research Centre, University of Malaya, Kuala Lumpur, Malaysia

**Keywords:** Alzheimer’s disease, acetylcholinesterase, butyrylcholinesterase, chalcones, molecular modelling

## Abstract

A series of C4-substituted tertiary nitrogen-bearing 2′-hydroxychalcones were designed and synthesised based on a previous mixed type acetylcholinesterase inhibitor. Majority of the 2′-hydroxychalcone analogues displayed a better inhibition against acetylcholinesterase (AChE) than butyrylcholinesterase (BuChE). Among them, compound **4c** was identified as the most potent AChE inhibitor (IC_50_: 3.3 µM) and showed the highest selectivity for AChE over BuChE (ratio >30:1). Molecular docking studies suggested that compound **4c** interacts with both the peripheral anionic site (PAS) and catalytic anionic site (CAS) regions of AChE. ADMET analysis confirmed the therapeutic potential of compound **4c** based on its blood–brain barrier penetrating. Overall, the results suggest that this 2′-hydroxychalcone deserves further investigation into the therapeutic lead for Alzheimer’s disease (AD).

## GRAPHICAL ABSTRACT

**Figure:**
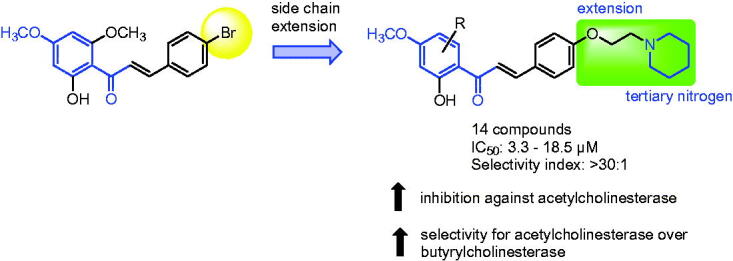

## Introduction

Alzheimer’s disease (AD) is the most typical form of dementia among older people which affects a person’s ability to carry out daily activities. About 47 million people live with dementia worldwide and this figure is anticipated to rise to more than 131 million by 2050^1^. It has been reported that loss of cholinergic transmission is one of the major causes of AD. Therefore, small molecule cholinesterase inhibitors that enhance cholinergic transmission can be used as a remedy for AD. One such example is donepezil hydrochloride (E2020, Aricept^®^), which is currently used in the palliative treatment for mild to moderate AD. Analysis of the crystal structure of donepezil in complex with human AChE suggested that the benzyl, piperidine and indanone rings of donepezil are important pharmacophores for binding to the active site^2^. Subsequently, a great deal of effort has been directed towards identifying potent AChE inhibitors based on the donepezil structure, involving structure-based design and new scaffolds^3–13^.

In our previous study, we have identified several 2′-hydroxychalcones that inhibited human AChE in the micromolar range and showed some selectivity for BuChE^14^. Kinetic studies indicated that these compounds were mixed-type inhibitors. Hence, the present study was undertaken to design and synthesise more potent 2′-hydroxychalcone derivatives. The synthesised derivatives were evaluated for their *in vitro* AChE and BuChE inhibitory activities. The Absorption, Distribution, Metabolism, Excretion and Toxicity (ADMET) parameters of these compounds were calculated. In addition, molecular modelling studies were carried out on the active compounds to gain further insight of the protein-ligand interactions.

## Materials and methods

### General

The chemicals and solvents used for synthesis were of analytical grade and were purchased from Sigma-Aldrich or Merck. Analytical thin-layer chromatography (TLC) was carried out on Merck precoated aluminium silica gel sheets (Kieselgel 60 F_254_). Column chromatography was performed with silica gel 60 (230–400 mesh) from Merck. UV/fluorescence was measured using Infinite^®^ M200 PRO multimode reader (Tecan Group Ltd., Männedorf, Switzerland). Melting points were taken on a Stuart melting point apparatus SMP30 (Staffordshire, UK) which has been certified for accuracy of ± 0.5 at 50 °C, ± 1.0 at 100 °C, ± 2.5 at 360 °C by the manufacturer. NMR spectra were obtained using Jeol ECA 400 (400 MHz) (Jeol Ltd., Akishima, Tokyo, Japan) or Bruker Avance III HD (600 MHz) (Bruker Biospin Corp., Billerica, MA). HRMS (ESI) analyses were performed using Agilent 1200 Series or Agilent 6530 Q-TOF (ESI) spectrometer with Agilent Zorbax C-18 column (Agilent Technologies, Santa Clara, CA). Human recombinant AChE and equine BChE were purchased from Sigma-Aldrich (St. Louis, MO).

### Synthesis of 2′-hydroxychalcone derivatives

The syntheses of designed compounds **4a–h** and **8a–f** are described in the supplemental data. Their purity was checked by HPLC, ^1^H and ^13^C-NMR spectroscopy and mass spectrometry. The ADMET parameters of the synthesised compounds were determined using Discovery Studio v3.1 (Accelrys Inc., San Diego, CA) and SwissADME^15^.

#### (*E*)-*N*,*N*-diethyl-2-(4-(3-(2-hydroxy-4-methoxyphenyl)-3-oxoprop-1-en-1yl)phenyl)acetamide (4a)

Yellow needle; yield 53%; mp 119.9–121.0 °C. ^1^H NMR (400 MHz, CDCl_3_): *δ* 13.38 (1H, s, OH), 7.78 (1H, d, *J* = 15.6 Hz, H_β_), 7.75 (1H, d, *J* = 8.0 Hz), 7.52 (2H, d, *J* = 8.0 Hz), 7.48 (1H, d, *J* = 15.6 Hz, H_α_), 7.25 (2H, d, *J* = 8.0 Hz), 6.40 (1H, dd, *J* = 8.0, 2.6 Hz), 6.39 (1H, d, *J* = 2.6 Hz), 3.78 (3H, s, OCH_3_), 3.66 (2H, s, –CH_2_CO), 3.32 (2H, q, *J* = 6.8 Hz, –NCH_2_–), 3.24 (2H, q, *J* = 7.0 Hz, –NCH_2_–), 1.06 (6H, m, –CH_3_); ^13^C NMR (100 MHz, CDCl_3_): *δ* 191.84 (C=O), 169.57 (C=O), 166.69, 166.23, 144.07, 138.44, 133.34, 131.26, 129.49, 128.81, 120.03, 114.11, 107.72, 101.10, 55.59 (OCH_3_), 42.43 (–NCH_2_–), 40.60 (–CH_2_CO), 40.32 (–NCH_2_–), 14.28 (–CH_3_), 12.93 (–CH_3_). HRMS calc. for [C_22_H_25_NNaO_4_]^+^ = [M + Na]^+^ = 390.1681, found = 390.1689.

#### (*E*)-*N*,*N*-diethyl-2-(4-(3-(2-hydroxy-4,5-dimethoxyphenyl)-3-oxoprop-1-en-1-yl)phenyl)acetamide (4b)

Yellow needles, yield 49%; mp 115.1–120 °C. ^1^H NMR (400 MHz, CDCl_3_): *δ* 13.31 (1H, s, OH), 7.80 (1H, d, *J* = 15.3 Hz, H_β_), 7.54 (2H, d, *J* = 8.0 Hz), 7.42 (1H, d, *J* = 15.5 Hz, H_α_), 7.26 (2H, d, *J* = 8.0 Hz), 7.18 (1H, s), 6.43 (1H, s), 3.86, 3.84 (3H each, s, OCH_3_), 3.68 (2H, s, –CH_2_CO), 3.33 (2H, m, –NCH_2_–), 3.27 (2H, m, –NCH_2_–), 1.07 (6H, m, –CH_3_); ^13^C NMR (100 MHz, CDCl_3_): *δ* 191.49 (C=O), 169.73 (C=O), 161.80, 157.14, 144.16, 141.99, 138.33, 133.43, 129.51, 128.84, 120.20, 112.02, 111.10, 100.85, 56.99 (OCH_3_), 56.21 (OCH_3_), 42.49 (–NCH_2_–), 40.45 (–CH_2_CO),*40.45 (–NCH_2_–),*14.26 (–CH_3_), 12.93 (–CH_3_). *Resonance signal overlapped. HRMS calc. for [C_23_H_27_NNaO_5_]^+^ = [M + Na]^+^ = 420.1787, found = 420.1798.

#### (*E*)-*N*,*N*-diethyl-2-(4-(3-(2-hydroxy-4,6-dimethoxyphenyl)-3-oxoprop-1-en-1-yl)phenyl)acetamide (4c)

Yellow needles; Yield 40%; mp 115.5–117.2 °C. ^1^H NMR (400 MHz, CDCl_3_): *δ* 14.23 (1H, s, OH), 7.79 (1H, d, *J* = 15.6 Hz, H_β_), 7.67 (1H, d, *J* = 15.6 Hz, H_α_), 7.47 (2H, d, *J* = 8.3 Hz), 7.22 (2H, d, *J* = 8.3 Hz), 6.01 (1H, d, *J* = 2.0 Hz), 5.87 (1H, d, *J* = 2.0 Hz), 3.83, 3.75 (3H each, s, OCH_3_), 3.35 (2H, s, –CH_2_CO), 3.31 (2H, q, *J* = 7.0 Hz, –NCH_2_–), 3.24 (2H, q, *J* = 7.0 Hz, –NCH_2_–), 1.05 (6H, m, –CH_3_); ^13^C NMR (100 MHz, CDCl_3_): *δ* 192.61 (C=O), 169.71 (C=O), 168.37, 166.24, 162.53, 141.99, 137.73, 134.13, 129.35, 128.61, 127.30, 106.36, 93.84, 91.26, 55.85 (OCH_3_), 55.56 (OCH_3_), 42.43 (–NCH_2_–), 40.58 (–CH_2_CO), 40.32 (–NCH_2_–), 14.27 (–CH_3_), 12.93 (–CH_3_). HRMS calc. for [C_23_H_27_NNaO_5_]^+^ = [M + Na]^+^ = 420.1787, found = 420.1788

#### (*E*)-1-(2-hydroxy-4-methoxyphenyl)-3-(4-(2-oxo-2-(pyrrolidin-1-yl)ethyl)phenyl)prop-2-en-1-one (4d)

Yellow needles; yield 45%; mp 139.2–141.7 °C. ^1^H NMR (400 MHz, CDCl_3_): *δ* 13.37 (1H, s, OH), 7.77 (1H, d, *J* = 16.0 Hz, H_β_), 7.74 (1H, d, *J* = 9.0 Hz), 7.51 (2H, d, *J* = 8.0 Hz), 7.47 (1H, d, *J* = 16.0 Hz, H_α_), 7.26 (2H, d, *J* = 8.0 Hz), 6.40 (1H, dd, *J* = 8.0, 2.4 Hz), 6.38 (1H, d, *J* = 2.4 Hz), 3.77 (3H, s, OCH_3_), 3.62 (2H, s, –CH_2_CO), 3.42 (2H, m, –NCH_2_–), 3.37 (2H, m, –NCH_2_–), 1.86 (2H, m, –CH_2_–), 1.77 (2H, m, –CH_2_–); ^13^C NMR (100 MHz, CDCl_3_): *δ* 191.83 (C=O), 168.57 (C=O), 166.68, 166.23, 144.07, 137.86, 133.37, 131.26, 129.76, 128.78, 120.03, 114.11, 107.71, 101.10, 55.59 (OCH_3_), 46.95 (–NCH_2_–), 46.05 (–NCH_2_–), 42.02 (–CH_2_CO), 26.13 (–CH_2_–), 24.35 (–CH_2_–). HRMS calc. for [C_22_H_23_NNaO_4_]^+^ = [M + Na]^+^ = 388.1525, found = 388.1526.

#### (*E*)-1-(2-hydroxy-4,5-dimethoxyphenyl)-3-(4-(2-oxo-2-(pyrrolidin-1-yl)ethyl)phenyl)prop-2-en-1-one (4e)

Yellow needles; yield 39%; mp 149.0–150.7 °C. ^1^H NMR (400 MHz, CDCl_3_): *δ* 13.31 (1H, s, OH), 7.81 (1H, d, *J* = 15.4 Hz, H_β_), 7.54 (2H, d, *J* = 8.0 Hz), 7.41 (1H, d, *J* = 15.5 Hz, H_α_), 7.29 (2H, d, *J* = 8.0 Hz), 7.18 (1H, s), 6.44 (1H, s), 3.87, 3.85 (3H each, s, OCH_3_), 3.63 (2H, s, –CH_2_CO), 3.41 (4H, m, –NCH_2_–), 1.86 (2H, m, –CH_2_–), 1.81 (2H, m, –CH_2_–); ^13^C NMR (100 MHz, CDCl_3_): *δ* 191.49 (C=O), 169.02 (C=O), 161.80, 157.13, 144.17, 141.99, 137.77, 133.47, 129.80, 128.81, 120.22, 112.02, 111.09, 100.84, 56.99 (OCH_3_), 56.21 (OCH_3_), 46.94 (–NCH_2_–), 46.29 (–NCH_2_–), 41.94 (–CH_2_CO), 26.11 (–CH_2_–), 24.43 (–CH_2_–). HRMS calc. for [C_23_H_24_NO_5_]^−^ = [M − H] ^−^ 394.1654, found = 394.1678.

#### (*E*)-2-(4-(3-(2-hydroxyphenyl)-3-oxoprop-1-en-1-yl)phenyl)acetic acid (4f)

Yellow needles; yield 61%; mp 194.1–194.6 °C. ^1^H NMR (400 MHz, DMSO-d_6_): *δ* 12.53 (1H, s, OH), 8.24 (1H, m), 8.01 (1H, d, *J* = 15.5 Hz, H_β_), 7.84 (2H, d, *J* = 8.0 Hz), 7.82 (1H, d, *J* = 15.5 Hz, H_α_), 7.54 (1H, m), 7.35 (2H, d, *J* = 8.0 Hz), 7.00 (1H, dd, *J* = 7.8, 2.4 Hz), 6.98 (1H, d, *J* = 2.4 Hz), 3.63 (2H, s, –CH_2_CO); ^13^C NMR (100 MHz, DMSO-d_6_): *δ* 194.09 (C=O), 172.75 (C=O), 162.33, 145.09, 138.71, 136.77, 133.35, 131.29, 130.54, 129.56, 121.84, 121.22, 119.64, 118.20, 41.03 (–CH_2_CO). HRMS calc. for [C_17_H_15_O_4_]^+^ = [M + H]^+^ = 283.0970, found = 283.0979.

#### (*E*)-2-(4-(3-(2-hydroxy-5-methoxyphenyl)-3-oxoprop-1-en-1-yl)phenyl)acetic acid (4g)

Orange powders; yield 44%; mp 91.7–93.6 °C. ^1^H NMR (400 MHz, CDCl_3_): *δ* 12.36 (1H, s, OH), 7.88 (1H, d, *J* = 15.6 Hz, H_β_), 7.60 (2H, d, *J* = 8.0 Hz), 7.55 (1H, d, *J* = 15.6 Hz, H_α_), 7.34 (2H, d, *J* = 8.0 Hz), 7.33 (1H, d, *J* = 2.5 Hz), 7.13 (1H, dd, *J* = 7.8, 2.5 Hz), 6.96 (1H, d, *J* = 8.0 Hz), 3.83 (3H, s, OCH_3_), 3.76 (2H, s, −CH_2_CO); ^13^C NMR (100 MHz, CDCl_3_): *δ* 193.46 (C=O), 170.65 (C=O), 158.01, 151.80, 145.32, 138.34, 133.30, 129.75, 129.08, 123.99, 120.02, 119.72, 119.44, 113.00, 56.25 (OCH_3_), 40.84 (–CH_2_CO). HRMS calc. for [C_18_H_17_O_5_]^+^ = [M + H]^+^ = 313.1071, found = 313.1036.

#### (*E*)-2-(4-(3-(2-hydroxy-4,6-dimethoxyphenyl)-3-oxoprop-1-en-1-yl)phenyl)acetic acid (4h)

Yellow powders; yield 58%; mp 139.2–141.2 °C. ^1^H NMR (400 MHz, CDCl_3_): *δ* 14.21 (1H, s, OH), 7.79 (1H, d, *J* = 15.6 Hz, H_β_), 7.68 (1H, d, *J* = 15.6 Hz, H_α_), 7.48 (2H, d, *J* = 8.0 Hz), 7.21 (2H, d, *J* = 8.0 Hz), 6.02 (1H, d, *J* = 2.2 Hz), 5.88 (1H, d, *J* = 2.2 Hz), 3.83, 3.76 (3H each, s, OCH_3_), 3.70 (2H, s, –CH_2_CO); ^13^C NMR (100 MHz, CDCl_3_): *δ* 192.60 (C=O), 169.01 (C=O), 168.38, 166.26, 162.53, 141.93, 137.53, 134.15, 129.25, 128.68, 127.35, 106.37, 93.85, 91.29, 55.88 (OCH_3_), 55.58 (OCH_3_), 40.79 (–CH_2_CO). HRMS calc. for [C_19_H_19_O_6_]^+^ = [M + H]^+^ = 343.1182, found = 343.1194.

#### (*E*)-1-(2-hydroxy-4-methoxyphenyl)-3-(4-(2-(piperidin-1-yl)ethoxy)phenyl)prop-2-en-1-one (8a)

Yellow solid; yield 67%; mp 166.7–169.7 °C. ^1^H NMR (600 MHz, CDCl_3_ (90%) and MeOH-*d*_4_ (10%)): *δ* 13.45 (1H, brs, OH), 7.76 (1H, d, *J* = 15.9 Hz, H_β_), 7.74 (1H, d, *J* = 8.0 Hz), 7.52 (2H, d, *J* = 8.0 Hz), 7.37 (1H, d, *J* = 15.4 Hz, H_α_), 6.87 (1H, d, *J* = 8.0 Hz), 6.40 (1H, d, *J* = 7.8 Hz), 6.39 (1H, s), 4.24 (2H, brt, –CH_2_–), 3.78 (3H, s, OCH_3_), 2.94 (2H, brt, –CH_2_–), 2.69 (4H, brs, –NCH_2_–), 1.70 (4H, brs, –CH_2_–), 1.46 (2H, brs,–CH_2_–); ^13^C NMR (150 MHz, CDCl_3_): *δ* 191.97 (C=O), 166.09, 165.97, 160.01, 143.98, 131.30, 130.35, 128.11, 118.27, 114.95, 114.02, 107.53, 100.96, 63.97 (–OCH_2_–), 56.70 (–CH_2_N–), 55.45 (–NCH_2_–), 54.37 (OCH_3_), 23.96 (–CH_2_–), 22.55 (–CH_2_–). HRMS calc. for [C_23_H_28_NO_4_]^+^ = [M + H]^+^ = 382.2018, found = 382.2036.

#### (*E*)-1-(2-hydroxy-4-methoxyphenyl)-3-(4-(2-(pyrrolidin-1-yl)ethoxy)phenyl)prop-2-en-1-one (8b)

Yellow needles; yield 72%; mp 189.9–192.1 °C. ^1^H NMR (600 MHz, CDCl_3_): *δ* 13.44 (1H, brs, OH), 7.77 (1H, d, *J* = 15.0 Hz, H_β_), 7.74 (1H, d, *J* = 9.0 Hz), 7.53 (2H, d, *J* = 8.5 Hz), 7.38 (1H, d, *J* = 15.4 Hz, H_α_), 6.89 (2H, d, *J* = 8.5 Hz), 6.42 (1H, dd, *J* = 9.0, 2.0 Hz), 6.39 (1H, s), 4.32 (2H, t, *J* = 5.0 Hz, –CH_2_–), 3.78 (3H, s, OCH_3_), 3.20 (2H, t, *J* = 5.0 Hz, –CH_2_–), 3.02 (4H, brs, –NCH_2_–), 1.95 (4H, brs, –CH_2_–); ^13^C NMR (150 MHz, CDCl_3_): *δ* 191.88 (C=O), 166.14, 166.10, 159.90, 143.91, 131.26, 130.41, 128.33, 118.46, 115.06, 114.12, 107.62, 101.06, 64.79 (–OCH_2_–), 55.58 (–CH_2_N–), 54.64 (OCH_3_), 54.25 (–NCH_2_–), 23.26 (–CH_2_–). HRMS calc. for [C_22_H_26_NO_4_]^+^ = [M + H]^+^ = 368.1862, found = 368.1885.

#### (*E*)-1-(2-hydroxy-4,5-dimethoxyphenyl)-3-(4-(2-(piperidin-1-yl)ethoxy)phenyl)prop-2-en-1-one (8c)

Dark red needles; yield 61%; mp 145.0–146.2 °C. ^1^H NMR (600 MHz, CDCl_3_): *δ* 13.37 (1H, s, OH), 7.78 (1H, d, *J* = 15.4 Hz, H_β_), 7.54 (2H, d, *J* = 8.6 Hz), 7.32 (1H, d, *J* = 15.5 Hz, H_α_), 7.20 (1H, s), 6.88 (2H, d, *J* = 8.6 Hz), 6.43 (1H, s), 4.38 (2H, t, *J* = 3.6 Hz, –CH_2_–), 3.86, 3.85 (3H each, s, OCH_3_), 3.20 (2H, t, *J* = 3.0 Hz, –CH_2_–), 2.99 (4H, brs, –NCH_2_–), 1.85 (4H, m, –CH_2_–), 1.54 (2H, brs, –CH_2_–); ^13^C NMR (150 MHz, CDCl_3_): *δ* 191.45 (C=O), 161.73, 159.75, 157.04, 143.96, 141.76, 130.44, 128.43, 118.53, 115.08, 112.04, 111.17, 100.84, 63.83 (–OCH_2_–), 57.05 (–CH_2_N–), 56.66 (–NCH_2_–), 56.19 (OCH_3_), 54.44 (OCH_3_), 23.61 (–CH_2_–), 22.51 (–CH_2_–). HRMS calc. for [C_24_H_30_NO_5_]^+^ = [M + H]^+^ = 412.2124, found = 412.2130.

#### (*E*)-1-(2-hydroxy-4,5-dimethoxyphenyl)-3-(4-(2-(pyrrolidin-1-yl)ethoxy)phenyl)prop-2-en-1-one (8d)

Orange solid; yield 58%; mp 167.0–169.0 °C. ^1^H NMR (600 MHz, CDCl_3_): *δ* 13.35 (1H, s, OH), 7.78 (1H, d, *J* = 15.4 Hz, H_β_), 7.55 (2H, d, *J* = 8.7 Hz), 7.34 (1H, d, *J* = 15.4 Hz, H_α_), 7.19 (1H, s), 6.90 (2H, d, *J* = 8.7 Hz), 6.44 (1H, s), 4.49 (2H, t, *J* = 4.5 Hz, –CH_2_–), 3.87, 3.86 (3H each, s, OCH_3_), 3.42 (2H, t, *J* = 4.6 Hz, –CH_2_–), 3.33 (4H, brs, –NCH_2_–), 2.09 (4H, brs, –CH_2_–); ^13^C NMR (150 MHz, CDCl_3_): *δ* 191.43 (C=O), 161.75, 159.37, 157.04, 143.84, 141.96, 130.49, 128.75, 118.74, 115.09, 112.03, 111.09, 100.85, 63.91 (–OCH_2_–), 57.03 (–CH_2_N–), 56.21 (–NCH_2_–), 54.55 (OCH_3_), 54.05 (OCH_3_), 23.27 (–CH_2_–). HRMS calc. for [C_23_H_28_NO_5_]^+^ = [M + H]^+^ = 398.1967, found = 398.1968.

#### (*E*)-1-(2-hydroxy-4,6-dimethoxyphenyl)-3-(4-(2-(piperidin-1-yl)ethoxy)phenyl)prop-2-en-1-one (8e)

Yellow needles; yield 69%; mp 164.3–166.7 °C. ^1^H NMR (600 MHz, CDCl_3_): *δ* 14.30 (1H, s, OH), 7.73 (1H, d, *J* = 15.6 Hz, H_β_), 7.68 (1H, d, *J* = 15.6 Hz, H_α_), 7.47 (2H, d, *J* = 8.4 Hz), 6.85 (2H, d, *J* = 8.4 Hz), 6.03 (1H, s), 5.89 (1H, s), 4.28 (2H, t, *J* = 5.0 Hz, –CH_2_–), 3.85, 3.76 (3H each, s, OCH_3_), 2.98 (2H, t, *J* = 5.0 Hz, –CH_2_–), 2.75 (4H, brs, –NCH_2_–), 1.74 (4H, m, –CH_2_–), 1.48 (2H, brs, –CH_2_–); ^13^C NMR (150 MHz, CDCl_3_): *δ* 192.55 (C=O), 168.36, 166.09, 162.47, 159.72, 142.13, 130.13, 128.92, 125.56, 114.98, 106.35, 93.83, 91.25, 64.61 (–OCH_2_–), 57.07 (–CH_2_N–), 55.87 (OCH_3_), 55.58 (–NCH_2_–), 54.60 (OCH_3_), 24.41 (–CH_2_–), 23.09 (–CH_2_–). HRMS calc. for [C_24_H_30_NO_5_]^+^ = [M + H]^+^ = 412.2124, found = 412.2131.

#### (*E*)-1-(2-hydroxy-4,6-dimethoxyphenyl)-3-(4-(2-(pyrrolidin-1-yl)ethoxy)phenyl)prop-2-en-1-one (8f)

Yellow solid; yield 70%; mp 157.0–159.0 °C. ^1^H NMR (600 MHz, CDCl_3_): *δ* 14.27 (1H, s, OH), 7.73 (1H, d, *J* = 15.5 Hz, H_β_), 7.67 (1H, d, *J* = 15.5 Hz, H_α_), 7.48 (2H, d, *J* = 8.6 Hz), 6.89 (2H, d, *J* = 8.6 Hz), 6.03 (1H, d, *J* = 2.3 Hz), 5.89 (1H, d, *J* = 2.3 Hz), 4.50 (2H, t, *J* = 4.4 Hz, –CH_2_–), 3.85, 3.77 (3H each, s, OCH_3_), 3.45 (2H, t, *J* = 4.4 Hz, –CH_2_–), 3.35 (4H, brs, –NCH_2_–), 2.10 (4H, brs, –CH_2_–); ^13^C NMR (150 MHz, CDCl_3_): *δ* 192.52 (C=O), 168.37, 166.17, 162.50, 158.75, 141.71, 130.18, 129.68, 126.10, 115.04, 106.35, 93.85, 91.28, 63.55 (–OCH_2_–), 55.89 (–CH_2_N–), 55.59 (OCH_3_), 54.54 (OCH_3_), 54.01 (–NCH_2_–), 23.26 (–CH_2_–). HRMS calc. for [C_23_H_28_NO_5_]^+^ = [M + H]^+^ = 398.1967, found = 398.1973.

### Enzyme inhibition studies

To determine the inhibitory activity of the target compounds towards cholinesterases, the AChE and BuChE inhibition assays were performed in 96 well plates by the method of Ellman with slight modifications^16^. First, sodium phosphate buffer (110 μL; pH 8.0) was added into each well followed by 20 μL of each test compound, 50 μL of 5,5′-dithiobis-(2-nitrobenzoic acid) (DTNB) (0.126 mM) and 20 μL of AChE or BuChE (0.15 units per mL). Then, the mixture was incubated for 20 min at 37 °C. Subsequently, the reaction was initiated by addition of 50 μL (0.120 mM) of the substrate, acetylthiocholine (ATC) iodide or butyrylthiocholine (BTC) iodide (depending on the enzyme). The hydrolysis of ATC or BTC was monitored with an Infinite^®^ M200 PRO multimode reader (Tecan Group Ltd., Männedorf, Switzerland) by measuring the absorbance of the yellow 5-thio-2-nitrobenzoate anion at 412 nm, every 30 s for 25 min. Donepezil, propidium iodide and tacrine were used as standard inhibitors. Each assay was performed in triplicate. The percentage of inhibition was calculated using the following expression:

(E–S)/E ×100

where E is the activity of the enzyme without test compound and S is the activity of the enzyme with the test compound. IC_50_ values were obtained from concentration-inhibition experiments by nonlinear regression analysis using PRISM^®^ v5.0 (GraphPad Inc., San Diego, CA).

### Molecular modelling studies

Three-dimensional structural models of the test compounds were built using Chem Bio-3D v13 (CambridgeSoft, Cambridge, MA) and saved in MOL2 format. The compounds were then prepared in a protonated form, where appropriate for physiological pH, and minimised using Hyperchem Pro v6.0 (geometry optimisation – MM^+^) to give the lowest energy conformation. The X-ray crystal structure of human AChE in complex with donepezil was retrieved from the Protein Data Bank (PDB ID: 4EY7)^2^. Ligand and water molecules were removed using Discovery Studio v3.1. Hydrogen atoms were added and double coordinates were corrected using Hyperchem Pro v6.0 (Hypercube Inc., Gainesville, FL). Hydrogen atoms were again added, non-polar hydrogen atoms were merged, and missing atoms were repaired using AutoDock Tools v4^17^. Gasteiger charges were added and AutoDock v4.0 type atoms were assigned to the protein. Then, test compounds were randomly docked into the protein structure with AutoDock v4.0 using a hybrid Lamarckian Genetic Algorithm^18^, with an initial population size of 150 and a maximum number of 2,500,000 energy evaluations. The grid box was set to cover the entire protein with a spacing of 0.375 Å. The root mean square deviation (RMSD) tolerance was set to 2.0 Å for the clustering of docked results and the docked pose of each ligand was selected on the basis of free energy of binding and cluster analysis. Ligand–protein interactions were analysed using Discovery Studio v3.1 and PoseView^19^.

## Results and discussion

### Design strategy

**Figure 1.**
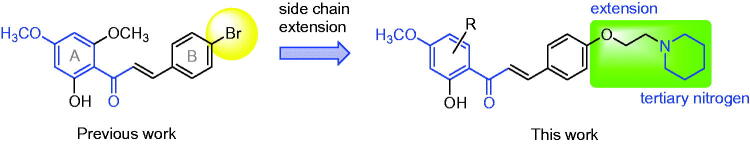
Design strategy for 2′-hydroxychalcones with modified C4-substituents.

Docking of the corresponding 2′-hydroxy-4′,6′-dimethoxy-4-bromochalcone (Figure 1) described in our previous work to the crystal structure of the human AChE (PDB ID: 4EY7) reveals room for a possible side-chain extension from the C4 position to engage extra binding interactions^14^. The docking structure suggests that the indole ring of Trp86 at the bottom of the active site gorge is a perspective binding site by π-cation interaction. Thus, we focussed on a well-established chain extension strategy which involves the incorporation of a tertiary nitrogen functional group linked into the C4 position of 2′-hydroxychalcone via a short alkyl chain^20^. We designed and synthesised a set of 14 target derivatives by varying substituent at the C4 position to increase the potency (Table 2, compounds **4a–h** and **8a–f**).

### Chemistry

**Scheme 1.**
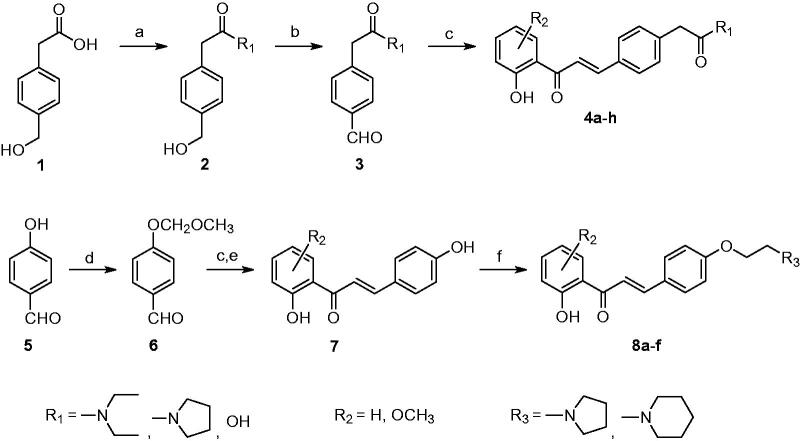
Syntheses of tertiary nitrogen-bearing 2′-hydroxychalcones. Reaction conditions: (a) EDC, HOBt, R_1_NH, CH_2_Cl_2_, 18 h, 28 °C (b) Dess-Martin periodinane, CH_2_Cl_2_, 18 h, 28 °C (c) 2′-hydroxyacetophenone, 50% aqueous NaOH, MeOH, 24 h, 28 °C (d) MOM-Cl, Et_3_N, CH_2_Cl_2_, 4 h, 28 °C (e) 1 M HCl, MeOH, 2 h, 60 °C (f) 1-(2-chloroethyl)piperidine⋅HCl or 1-(2-chloroethyl)pyrrolidine⋅HCl, Cs_2_CO_3_, NaI, acetone, 24 h, 60 °C.

The syntheses of target derivatives are outlined in Scheme 1. The amide coupling between 4-(hydroxymethyl)phenylacetic acid **1** and the corresponding amine was conducted under the standard carbodiimide chemistry afforded compound **2**, which was further oxidised to benzaldehyde **3** with periodinane. The 2′-hydroxychalcone derivatives **4a–h** were obtained from the Claisen-Schmidt condensation of different substituted 2′-hydroxyacetophenones and benzaldehyde **3** in the presence of NaOH in methanol^14^. Compound **7** was synthesised in a similar fashion by using different substituted 2′-hydroxyacetophenones and methoxymethyl ether (MOM) protected benzaldehyde **6**. Finally, compounds **8a–f** were generated from compound **7** with 1-(2-chloroethyl)piperidine hydrochloride or 1-(2-chloroethyl)pyrrolidine hydrochloride in the presence of Cs_2_CO_3_ and NaI^21^. All target compounds **4a–h** and **8a–f** were purified by silica gel chromatography. Their structures were confirmed by using nuclear magnetic resonance spectroscopy (^1^H NMR, ^13^C NMR) and high-resolution mass spectrometry (HRMS). Representative spectroscopic spectra are provided in the supplemental data.

### ADMET parameters

The predicted ADMET parameters of designed compounds **4a–h** and **8a–f** are summarised in Table 1. According to the ADMET analysis, all the compounds follow Lipinski’s rule by causing no violation^22–24^. Compounds **4g** and **4h** are predicted by the SwissADME to be unable to cross the blood–brain barrier^15^. Despite the designed compounds are moderately soluble in water, they have a high degree of gastrointestinal absorption. This may be due to the presence of tertiary nitrogen atom in these compounds that allow the formation of salt, resulting in improved solubility and gastrointestinal adsorption.

**Table 1.** ADMET parameters for 2′-hydroxychalcones **4a–h** and **8a–f**.

Compound	MW	HBD	HBA	Log P	PSA(Å^2^)	AS	GIA	BBB
**4a**	367.44	1	4	3.59	67.70	Moderate	High	Yes
**4b**	397.46	1	5	3.57	76.63	Moderate	High	Yes
**4c**	397.46	1	5	3.57	76.63	Moderate	High	Yes
**4d**	365.42	1	4	3.35	67.70	Moderate	High	Yes
**4e**	395.45	1	5	3.34	76.63	Moderate	High	Yes
**4f**	282.29	2	4	3.12	76.23	Soluble	High	Yes
**4g**	312.32	2	5	3.11	85.16	Soluble	High	No
**4h**	342.34	2	6	3.09	94.10	Soluble	High	No
**8a**	381.46	1	5	4.48	59.33	Moderate	High	Yes
**8b**	367.44	1	5	4.03	59.33	Moderate	High	Yes
**8c**	411.49	1	6	4.47	68.26	Moderate	High	Yes
**8d**	397.46	1	6	4.01	68.26	Moderate	High	Yes
**8e**	411.49	1	6	4.47	68.26	Moderate	High	Yes
**8f**	397.46	1	6	4.01	68.26	Moderate	High	Yes
Donepezil	379.49	0	4	4.57	38.51	Moderate	High	Yes
Propidium	414.59	2	2	3.40	58.43	Moderate	High	No
Tacrine	198.26	1	2	2.79	37.80	Moderate	High	Yes

MW: molecular weight; HBD: number of hydrogen bond donors; HBA: number of hydrogen bond acceptors; log P: logarithm of octanol–water partition coefficient; PSA: polar surface area. AS: aqueous solubility; GIA: gastrointestinal absorption; BBB: blood–brain barrier.

### Cholinesterase inhibitory activity

The designed 2′-hydroxychalcone derivatives were evaluated for their *in vitro* inhibitory activities against human recombinant AChE according to Ellman’s colorimetric assay method^16^. The results are summarised in Table 2. Most of the tested compounds showed promising AChE inhibitory activity when screened at 10 µM. Based on the structure–activity relationship analysis, it is clear that AChE inhibitory activity was closely related to the C4-substituent. Generally, the tertiary amine substituted compounds possessed better AChE inhibitory activity than that of tertiary amide substituted compounds. The results of **8a**, **8 b**, and **8d–f** demonstrated that compounds with piperidine or pyrrolidine substituted at C4 position were comparably potent as that of donepezil and tacrine against AChE. When examining the efficacy of compounds **4a–e**, the presence of 6′-methoxy substituent in the A ring favour the AChE activity. Compounds **4f–h** which lack of a tertiary nitrogen substituent in B ring were inactive, indicating that the carboxylic group is not favoured for interaction. Besides, the length of the C4 side chain also played a significant role in the AChE inhibitory activity. Tertiary amine-bearing derivatives **8a–f** which contain a side chain of 2 carbon atoms, displayed a better inhibition than those derivatives without side chain described in our previous work^14^.

Subsequently, IC_50_ experiments were carried out for compounds **4c**, **8a**, **8 b**, and **8d–f** against human AChE and equine BuChE. The latter is considered a good model for human BuChE due to its high sequence similarity to the human form (90% sequence identity)^25^. The IC_50_ values are included in Table 3. Generally, these compounds showed higher activity towards AChE with IC_50_ values in the range of 3–19 µM. Among the target derivatives, compound **4c** was the most active towards AChE with IC_50_ = 3.3 µM. Moreover, compound **4c** exhibited the highest selectivity for AChE over BuChE (SI >30). High AChE selectivity over BuChE (SI >24) was also observed for compound **8a**, which possesses a piperidinyl ethyl ether substituted at the C4 position. Studies have shown that high AChE selectivity over BuChE may reduce the associated side effects of AD treatment^26^. Hence, compounds with high AChE selectivity over BuChE may be clinically useful. As can be seen in Table 3, the designed compounds generally showed lower BuChE inhibitory activity as compared to that of AChE. Compound **8e** was the most active towards BuChE with IC_50_ = 18.3 μM. In comparison, compound **8a** which lack of a 6′-methoxy substituent in the A ring was inactive, indicating that the 6′-methoxy group is important for interaction.

**Table 2.** Substituent patterns and human acetylcholinesterase inhibitory activity of 2′-hydroxychalcones **4a–h** and **8a–f**.

Compound	Structure	Human AChE (% Inhibition at 10 μM)^a^
**4a**	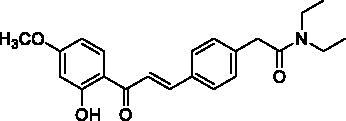	67.2
**4b**	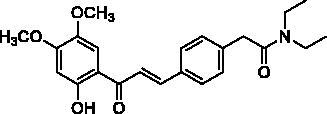	58.5
**4c**	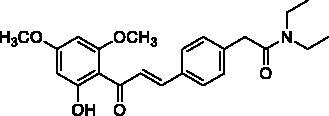	87.5
**4d**	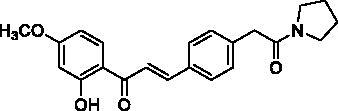	30.8
**4e**	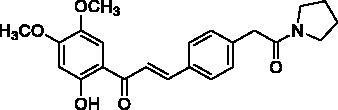	58.9
**4f**	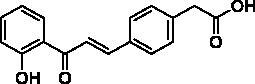	10.7
**4g**	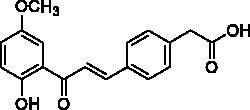	27.0
**4h**	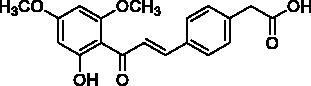	15.6
**8a**	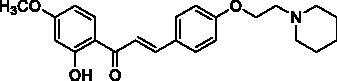	79.9
**8b**	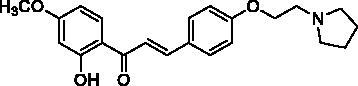	83.0
**8c**	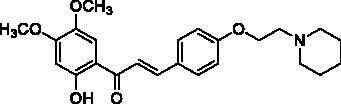	52.5
**8d**	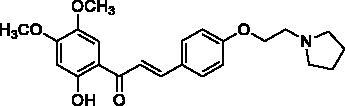	80.6
**8e**	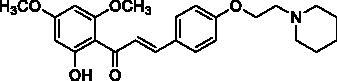	83.6
**8f**	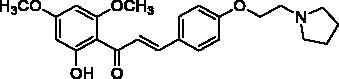	81.4
Donepezil^b^		92.0
Propidium^c^		52.0
Tacrine^d^		89.0

^a^Values are expressed as mean of triplicate; ^b^standard PAS and CAS-binding AChE inhibitor; ^c^standard PAS-binding AChE inhibitor; ^d^standard CAS-binding AChE inhibitor.

**Table 3.** Human AChE and equine BuChE inhibitory activities of the most active tertiary nitrogen containing 2′-hydroxychalcones.

Compound	Human AChE IC_50_/µM^a^	Equine BuChE IC_50_/µM^a^	Selectivity index^b^
**4c**	3.3 ± 1.3	>100	>30
**8a**	4.2 ± 1.3	>100	>24
**8b**	12.8 ± 4.8	65.6 ± 14.8	5.1
**8d**	15.9 ± 1.8	89.9 ± 12.0	5.7
**8e**	4.6 ± 0.9	18.3 ± 8.0	4.0
**8f**	18.5 ± 3.8	31.3 ± 8.2	1.7
Donepezil^c^	0.09 ± 0.01	1.9 ± 0.2	21
Propidium^d^	11 ± 3	20.8 ± 2.1	1.9
Tacrine^e^	0.19 ± 0.04	0.011 ± 0.001	0.06

^a^Values are expressed as mean of triplicate; ^b^Selectivity index = IC_50_ (BuChE)/IC_50_ (AChE); ^c^Standard PAS and CAS-binding AChE inhibitor; ^d^Standard PAS-binding AChE inhibitor; ^e^Standard CAS-binding AChE inhibitor.

### Molecular docking

The most active compounds **4c**, **8a**, and **8e** were docked into an AChE model obtained from the X-ray crystal structure of human AChE in complex with donepezil (PDB ID: 4EY7)^2^ in order to study the protein–ligand binding interactions.

**Figure 2.**
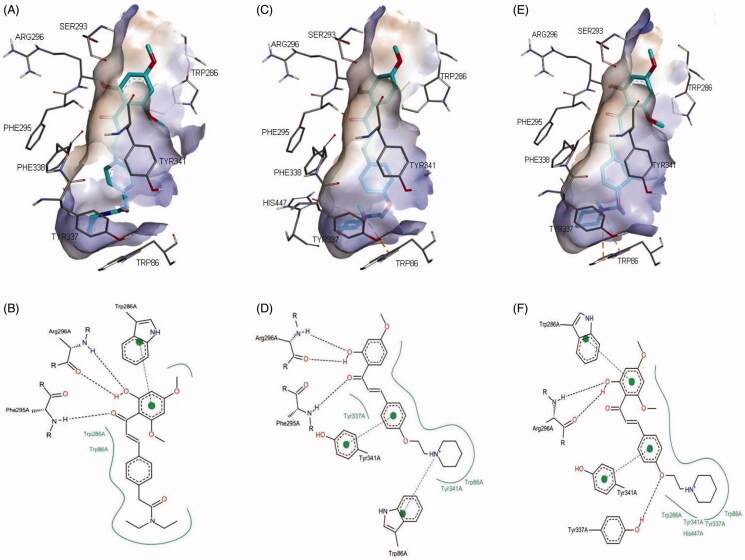
Representations of compounds **4c** (A, B), **8a** (C, D), and **8e** (E, F) in complex with human AChE (PDB ID: 4EY7). (A, C, and E) 3D representations of the binding pose. The hydrophobic surfaces of the interacting residues are shown in blue relief. Hydrogen bonds and π–cation interactions are depicted with green and orange dotted lines, respectively; (B, D, and F) Schematic representations of the binding interactions. Hydrogen bonds and π–π stacking interactions are depicted with black and green dotted lines, respectively. The green curve represents other non-polar interactions.

All docked compounds were found to exhibit binding poses similar to those of donepezil occupying both the peripheral anionic site (PAS) and catalytic anionic site (CAS) of the AChE (Figure 2). It was observed that the aromatic ring A of compounds **4c** and **8e** formed π–π stacking interactions with the PAS residue, Trp286, whereas aromatic ring B of compound **8a** formed π–π stacking interactions with Tyr341 (at the entrance to the gorge leading to that CAS). In the case of compounds **4c** and **8a**, the carbonyl oxygen atom and the *ortho*-hydroxyl group on the A ring were observed to form hydrogen bonds with backbone atoms from acyl pocket residues, Phe295 and Arg296, respectively. This may be one of the reasons to account for the greater activity of these two compounds, as compound **8e** only interacted with Arg296. The positively-charged nitrogen atom of the piperidine ring in compound **8a** formed π–cation interaction with CAS site aromatic residue, Trp86. This interaction is particularly important as several studies have reported its implication for biological recognition of human AChE^27^^,^^28^. In the case of compound **8e**, an extra hydrogen bonding interaction was formed between the ether oxygen atom and residue Tyr337. Taken together, these interactions have contributed to the enhanced potency of 2′-hydroxychalcone with modified C4-substituents. The docking findings are in agreement with the experimental results and provide reasons for improved cholinesterase inhibitory activity of the designed derivatives.

## Conclusions

In conclusion, novel 2′-hydroxychalcone derivatives with modified C4-substituents were designed, synthesised and evaluated as inhibitors of human AChE. Compounds with methoxy substituents on the A ring and piperidinyl ethyl ether substituent on B ring were generally found to be more potent. Molecular docking studies have suggested that these compounds interact with both the PAS and CAS regions of AChE. The most active 2′-hydroxychalcones showed high AChE selectivity over BuChE, suggesting that these compounds may be suitable to be developed into clinically useful drug candidates.

## Authors’ contributions

S.D.S: biological evaluation, chemical synthesis, molecular modelling, and manuscript drafting. T.J.T, S.B.N.: chemical synthesis. C.F.C., M.J.C.B.: project design, data analysis, manuscript preparation and revision. S.N.A.B.: interpretation of data and manuscript revision. R.O., N.A.R, and V. S. L: Project conception, design and co-ordination. All authors have read and approved the final version of the manuscript.

## Supplementary Material

Supplemental Material

## Data Availability

The raw data supporting the conclusions of this manuscript will be made available by the authors, without undue reservation, to any qualified researcher.
