# Supplementary material for: Analogues of 2′-hydroxychalcone with modified C4-substituents as the inhibitors against human acetylcholinesterase
Source: J Enzyme Inhib Med Chem. 2020 Nov 26;36(1):130–7. doi: 10.1080/14756366.2020.1847100 (PMC7822063; doi:10.1080/14756366.2020.1847100)

Supplemental data

Analogues of 2′-hydroxychalcone with modified C4-substituents as the inhibitors against human acetylcholinesterase

Sri Devi Sukumaran[1], Shah Bakhtiar Nasir[2], Jia Ti Tee[2], Michael J. C. Buckle[1], Rozana Othman[1], Noorsaadah Abd. Rahman[2], Vannajan Sanghiran Lee[2], Syed Nasir Abbas Bukhari[3], Chin Fei Chee[4,*],

[1]Department of Pharmacy, Faculty of Medicine, University of Malaya, 50603 Kuala Lumpur, Malaysia

[2]Department of Chemistry, Faculty of Science, University of Malaya, 50603 Kuala Lumpur, Malaysia

[3]College of Pharmacy, Jouf University, Al-Jouf, Kingdom of Saudi Arabia

[4]Nanotechnology and Catalysis Research Centre, University of Malaya, 50603 Kuala Lumpur, Malaysia

Representative spectroscopic spectra: page 2-7

^{1}H- and ^{13}C-NMR spectra of **8a**

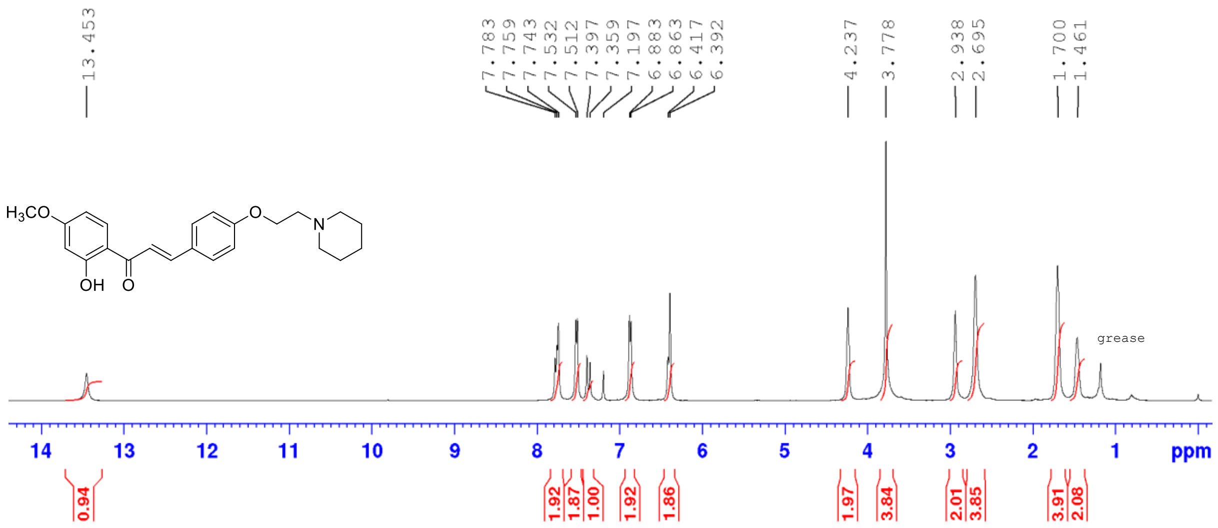

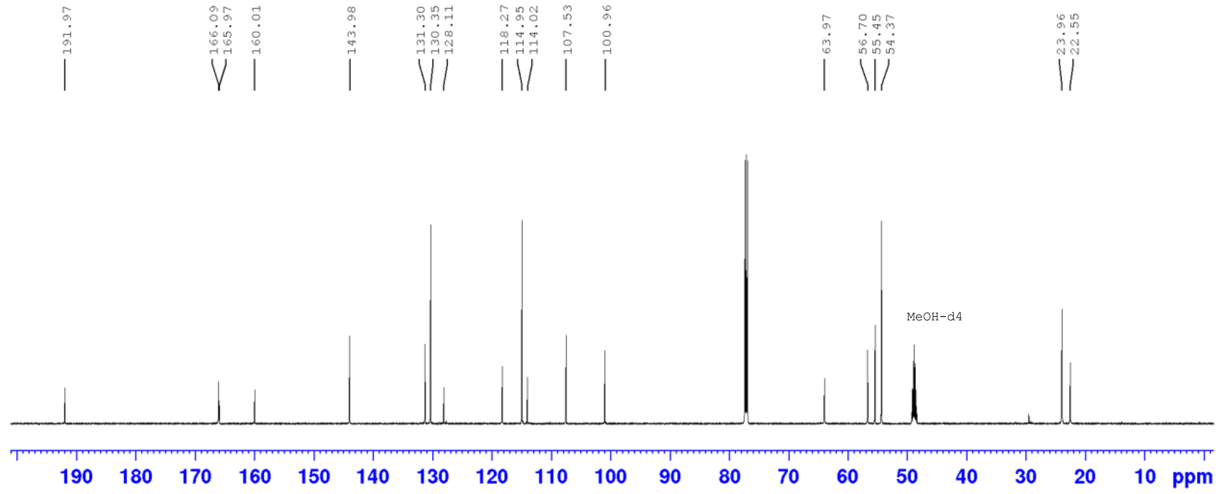

^{1}H- and ^{13}C-NMR spectra of **8b**

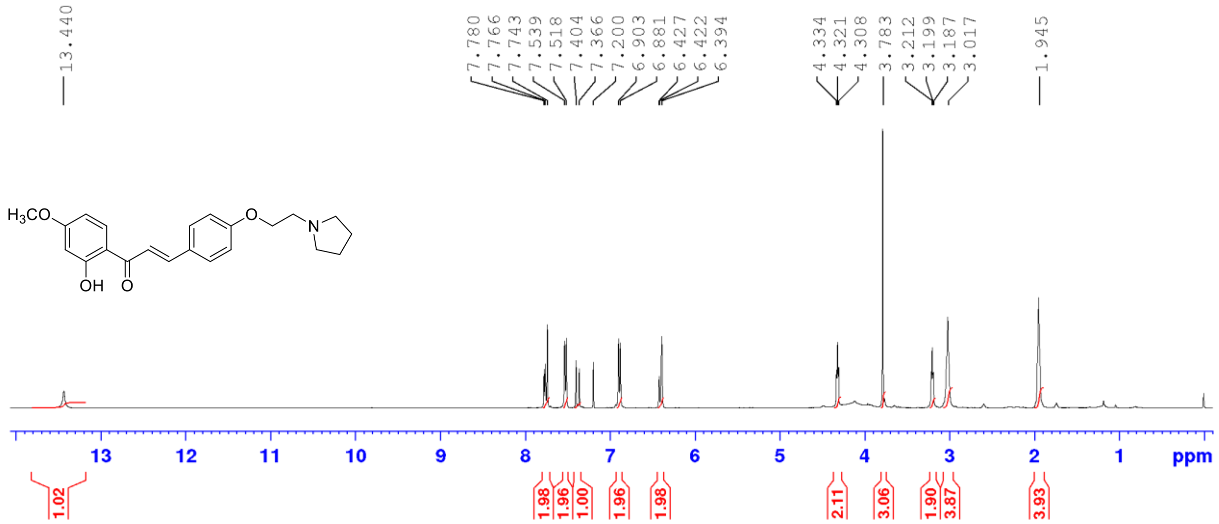

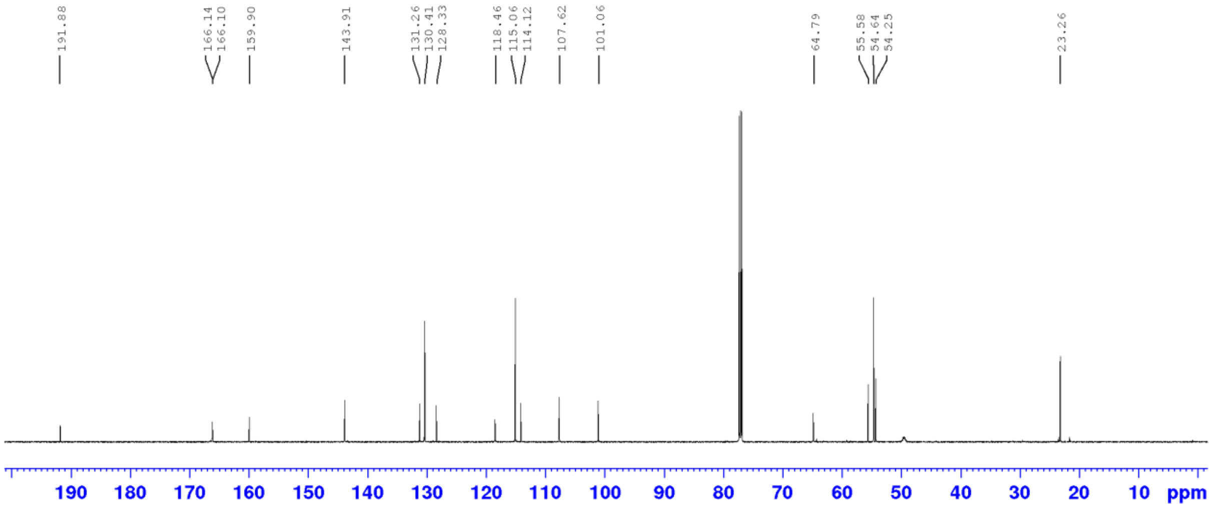

^{1}H- and ^{13}C-NMR spectra of **8c**

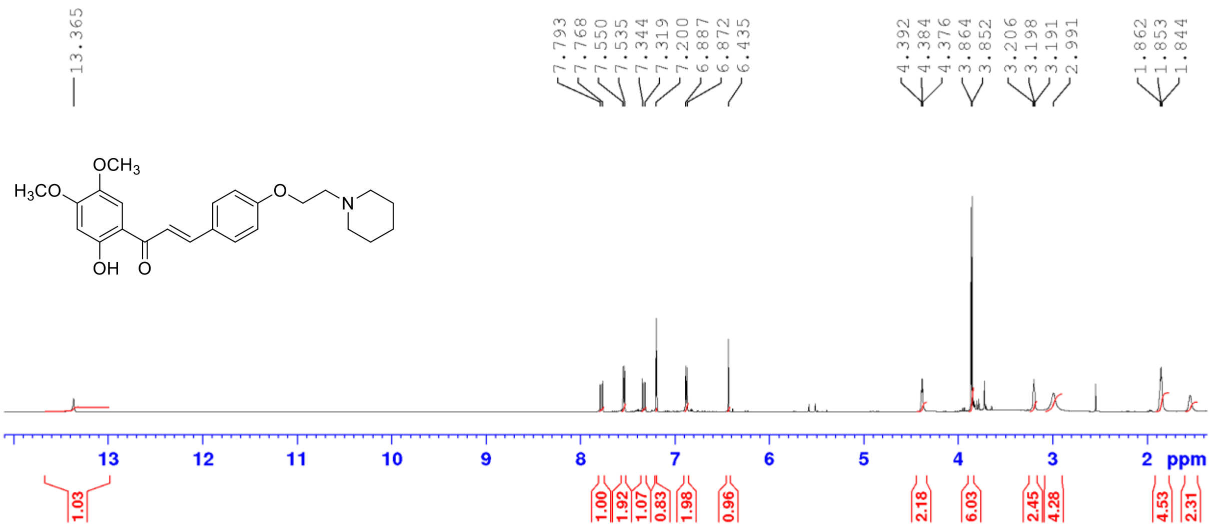

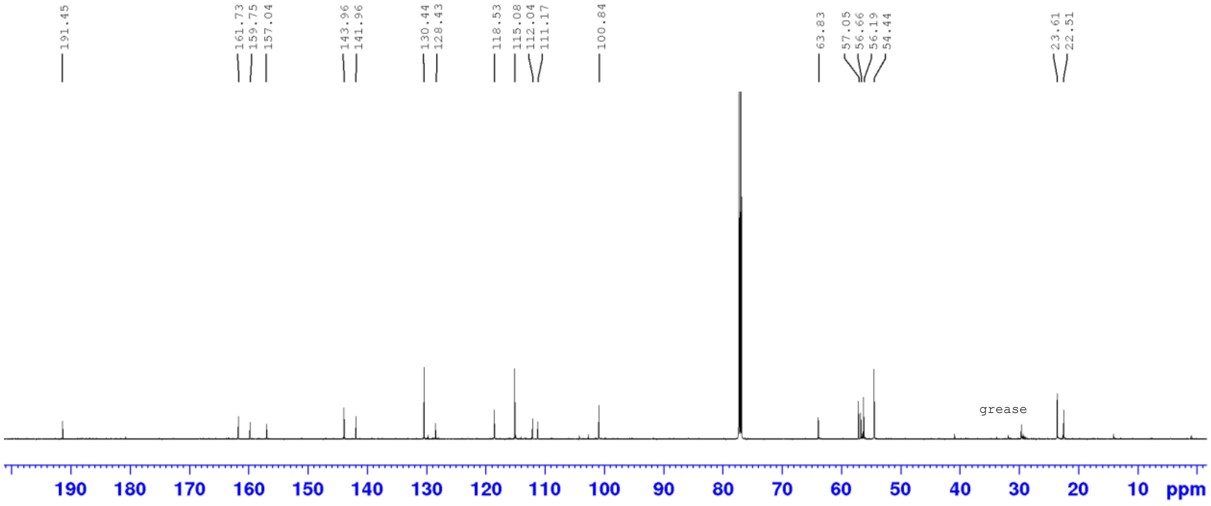

^{1}H- and ^{13}C-NMR spectra of **8d**

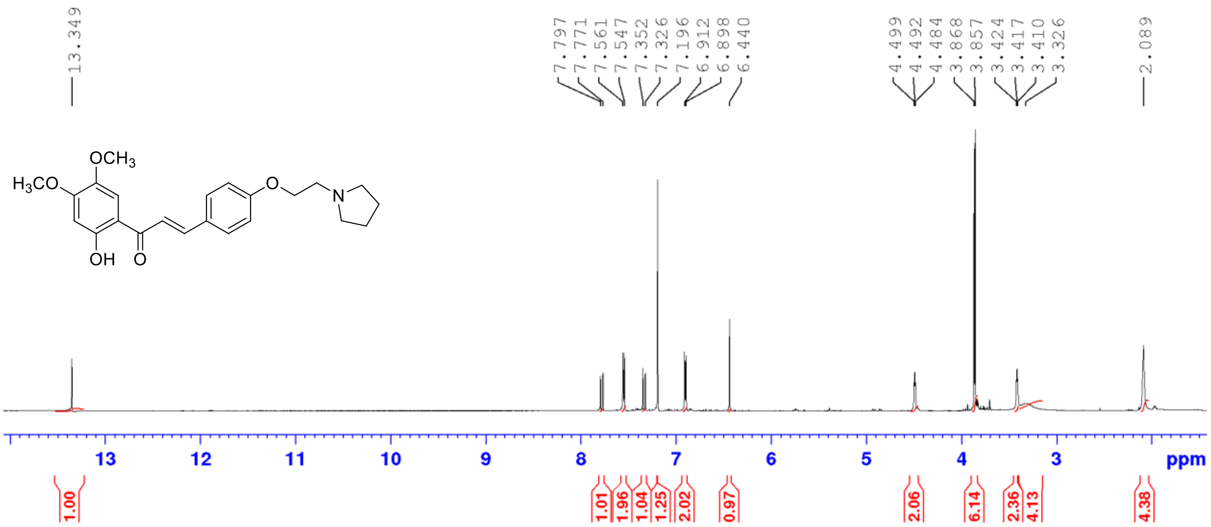

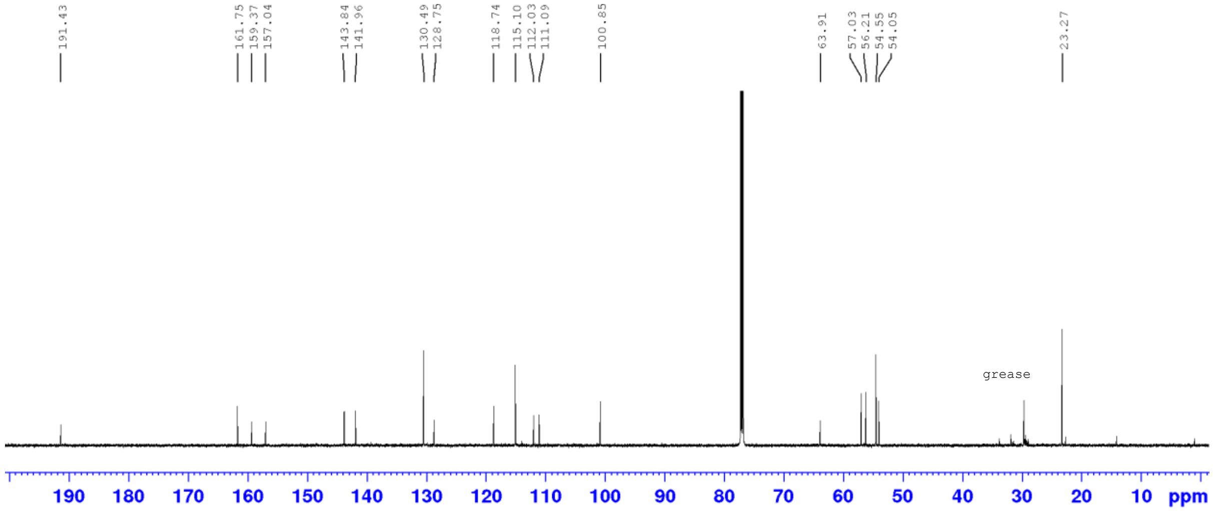

^{1}H- and ^{13}C-NMR spectra of **8e**

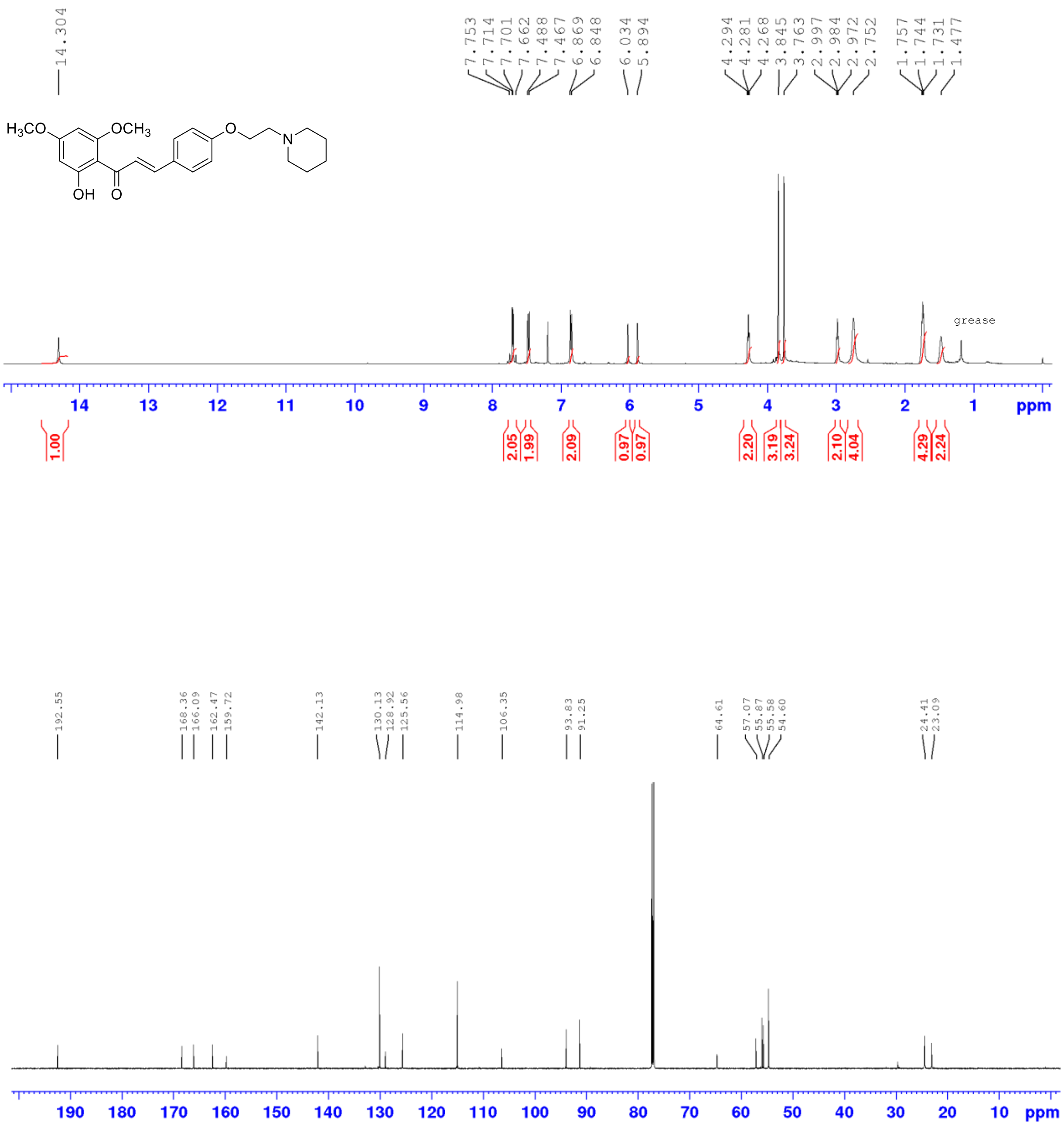

^{1}H- and ^{13}C-NMR spectra of **8f**

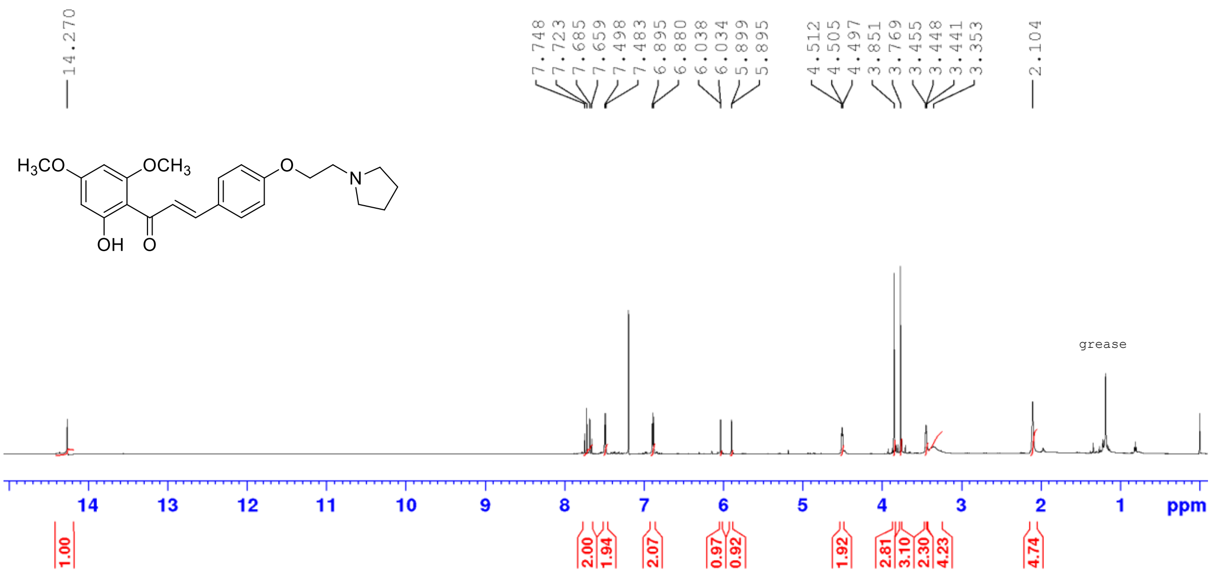

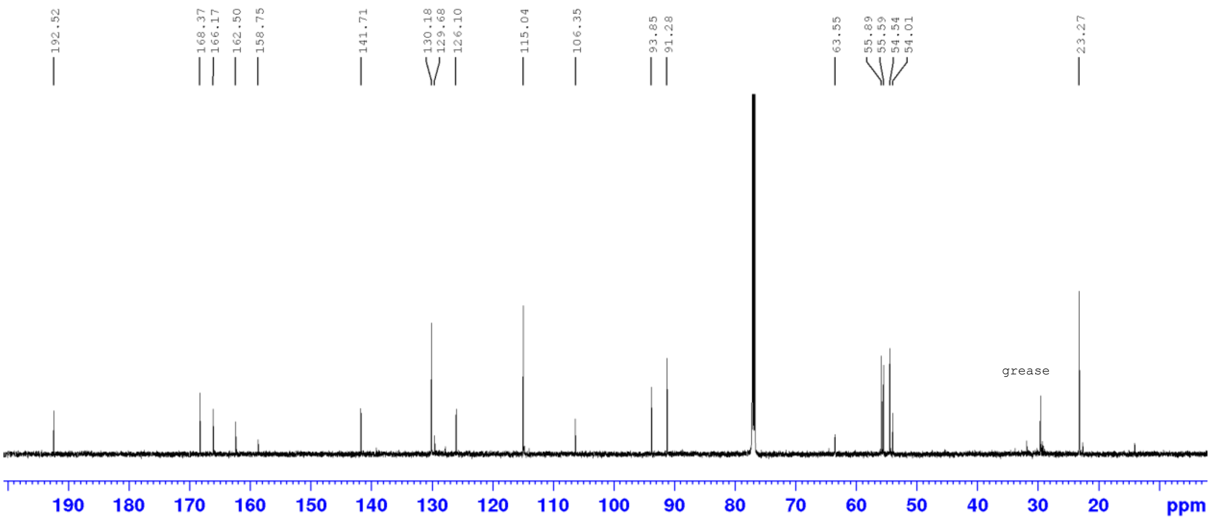

Supplement: Supplemental Material [file IENZ_A_1847100_SM6445.pdf]
